# Results of a population-based-assessment: we need better communication and more profound patient involvement

**DOI:** 10.1007/s00508-016-1165-8

**Published:** 2017-01-18

**Authors:** Gerald Sendlhofer, Gudrun Pregartner, Karina Leitgeb, Magdalena Hoffmann, Andrea Berghold, Christian Smolle, Gernot Brunner, Lars Peter Kamolz

**Affiliations:** 1 Executive Department for Quality and Risk Management, University Hospital Graz, Auenbruggerplatz 1, 8036 Graz, Austria; 2 Research Unit for Safety in Health, Division of Plastic, Aesthetic and Reconstructive Surgery, Department of Surgery, Medical University of Graz, Graz, Austria; 3 Austrian Society for Quality and Safety in Healthcare (ASQS), Graz, Austria; 4 Institute for Medical Informatics, Statistics and Documentation, Medical University of Graz, Graz, Austria; 5 University Hospital Graz, Graz, Austria

**Keywords:** Patient safety, Perception, Survey, Communication, Patient involvement

## Abstract

**Background:**

In Austria several regulations were published in order to support initiatives to increase patient safety. Since then, many patient safety projects were implemented in Austrian hospitals; therefore, it was the aim of the current survey to examine the perceptions of Austrian citizens with respect to topics relevant to patient safety.

**Methods:**

Between 8 and 22 October 2015 a qualitative cross-sectional telephone interview study was performed. A sample of citizens above 14 years of age was randomly drawn. The survey contained 6 questions. In each of the nine states of Austria, a representative number of citizens were interviewed.

**Results:**

In total 1021(female: 52.3%) telephone interviews were performed and 249 (24.7%) citizens stated that trust/confidence in patient safety is very high, 571 (55.9%) assessed the reputation of a hospital as very important and 739 (72.4%) stated that a detailed explanation of the treatment as well as information on associated risk factors and possibilities of further treatments is very important. Of the respondents 722 (70.7%) stated that patient safety measures in a given hospital are very important, 807 (79.0%) stated that it is important to be informed about patient safety measures and 547 (53.6%) stated that if something did not satisfactorily function they would complain to the hospital. Significant differences occurred for states with and without university hospitals.

**Conclusion:**

The results of the survey give cause for concern as the majority of interviewed citizens have medium or low trust/confidence in patient safety. Furthermore, more than two-thirds of Austrian citizens revealed that detailed explanation of treatment, information on associated risk factors, information about patient safety measures to predict medical errors and information about patient safety measures which are in place in a hospital are very important. The study showed that patient safety is an important topic for Austrian citizens and they want to be informed and involved. The study also indicated the need to promote patient safety aspects and to decrease the number of people who are not confident concerning patient safety in Austrian hospitals.

## Introduction

Since the report *To Err is Human* in 1999 patient safety initiatives all over the world became an integral part in healthcare systems [1, 2]. For example, in Austria several regulations were published in order to support initiatives to increase patient safety: In 2005, the Health Care Quality Act was released [3] followed by a quality strategy [4] and a nationwide patient safety strategy [5]. The overall aim of the safety strategy was to increase patient safety, empower patients to actively take part in healthcare processes and to inform citizens about patient safety concerns. Citizens and patients should be health literate with regard to patient safety issues [5]. According to these regulations in Austrian hospitals several patient safety projects were initiated and the main focus lay on implementation of clinical risk management, safe surgery or measures to increase hygiene aspects [6–8]; however, as a next step to further increase patient safety, as it was already aimed in one of these regulations, it is also necessary to involve patients in quality and safety initiatives, which is a mostly unexploited resource so far [9, 10]. Patients can speak up when they are concerned about their safety and can thereby help to prevent medical errors [9]. This is to a certain point a paradigmatic shift because patients move from being passive recipients to active participants [11]. In 2006, a telephone interview on discharged patients from a hospital in the United States of America (USA) showed that 91% of discharged patients agreed that they could help to prevent medical errors [12]. Patients are willing to help preventing errors but in order to do so they also have to be informed about patient safety standards [9, 12].

In 2010, according to the Special Eurobarometer on patient safety and quality of healthcare Austrian patients were highly trusting in hospital care [13]. Whilst in Greece 83% perceived that patients could be harmed by hospital care, in Austria 79% of respondents felt safe. It was obvious that countries with high levels of expenditure for a social health insurance system showed highest levels of satisfaction [14]. Asked for specific adverse events when receiving healthcare, such as i) hospital infections, ii) incorrect, missed or delayed diagnosis, iii) medication related errors, iv) surgical errors and v) medical device or related equipment errors, Austria had the highest proportion of those who perceived that these adverse events will not occur [13]; however, in the case of an adverse event 57% of Austrians would report the event [13].

In 1999, experts estimated that approximately 98,000 people die in any given year from medical errors that occur in US hospitals [1]. A recently published analysis showed an even worse picture and assumed that medical error is now the third leading cause of death in US hospitals [15]. Considering the overall importance of patient safety and patient willingness to participate in order to prevent medical errors, a survey was performed in Austria. The study primarily aimed to examine the perceptions of Austrian citizens with respect to patient safety relevant topics. The secondary aim was to evaluate if varying demands emerge with respect to sex, age, income and regional differences.

## Material and methods

According to the ethical committee of the Medical University of Graz there is no legal requirement for an ethical vote as the survey did not include patients or employees of the respective organization.

### Survey

Between 8 and 22 October 2015 a qualitative cross-sectional telephone interview study was performed. A sample of 1021 citizens above 14 years of age was drawn randomly by Das Österreichische Gallup-Institut. The survey contained six questions (Table 1). In each of the nine states of Austria, a representative number of citizens were interviewed including information on sex, age, income and origin. Informed consent was obtained by asking interviewed citizens if they would like to participate to the respective telephone survey. Citizens who denied participation were not interviewed.

**Table 1:** Questions contained in the survey

Number	Question
1	How important are the following aspects to you, in relation to a hospital? – very important, fairly important, slightly important, not at all important, no opinion
1.1	– Reputation of the hospital
1.2	– Detailed explanation of the treatment as well as information on associated risk factors and possibilities of further treatment
1.3	– Patient safety measures, such as patient identification by means of patient wristbands, safe surgery checklists or hand hygiene
2	What level of trust/confidence do you place in patient safety within Austrian health system? – very high, high, medium, low, no opinion
3	Is it important to you to be informed about patient safety measures in your hospital (relating to standards on hygiene, patient identification, safe surgery and drug safety, etc.)? – yes, no, no opinion
4	If you were of the opinion that something did not satisfactorily function (e. g. lack of empathic and respectful communication, lack of information on treatment, or complications) would you be willing to make a written complaint? – yes, no, no opinion

According to the population census 2015 by Statistik Austria, Austria has 8,579,000 citizens (100%), thereof in Vienna 1,794,800 (20.9%), Lower Austria 1,636,300 (19.1%), Upper Austria 1,436,800 (16.7%), Styria 1,221,000 (14.2%), Tyrol 728,500 (8.5%), Carinthia 557,400 (6.5%), Salzburg 538,300 (6.3%), Vorarlberg 378,500 (4.4%) and Burgenland 288,200 (3.4%) [16].

### Statistical analysis

Survey data were analyzed using descriptive statistics for the total cohort and for each of the subgroups by sex, age, income and state. Categorical variables are presented as absolute and relative frequencies. To assess differences between the subgroups the following statistical tests were applied according to subgroup factor and answer type: questions of ordinal answer type (1.1–2) were analyzed using the Mann-Whitney U‑test for binary factors (sex, state with university clinic) or the Jonckheere-Terpstra test for ordinal factors (age, income); questions of binary answer type (3–4) were analyzed using Fisher’s exact test for binary factors or the Cochran-Armitage test for ordinal factors. The answer categories “no opinion” and “not specified” were considered as missing values for the purpose of the tests. Since the study was not hypothesis-driven, all analyses are of a purely exploratory nature. All analyses were conducted using R version 3.2.2.

## Results

### Sample characteristics

Due to regional differences with respect to population density in each of the nine states in Austria, the percentage of interviewed citizens per state was carefully chosen and corresponded to the percentage of citizens living in each of the nine states. In total 1021 (female: 534, 52.3%) telephone interviews were performed, thereof 21.5% in Vienna, 18.5% in Lower Austria, 17.6% in Upper Austria, 15.8% in Styria, 7.9% in Carinthia, 7.5% in Tyrol, 5.7% in Salzburg, 2.9% in Burgenland and 2.4% in Vorarlberg. Of these, 516 (50.5%) citizens lived in states having a university hospital (Vienna, Styria, Salzburg and Tyrol; Upper Austria was excluded as the university hospital was established in 2016), 234 (22.9%) citizens were between 14 and 30 years of age, 404 (39.6%) were between 31 and 50 years of age and 383 (37.5%) were older than 50 years of age. Concerning income, 182 (17.8%) citizens reported earning less than 1500 €, 215 (21.1%) earned between 1500 and 2400 €, 125 (12.2%) earned between 2400 and 3000 €, 271 (26.5%) earned more than 3000 € income per month and 228 (22.3%) did not provide any information.

### Importance of reputation of a hospital

**Fig. 1:**
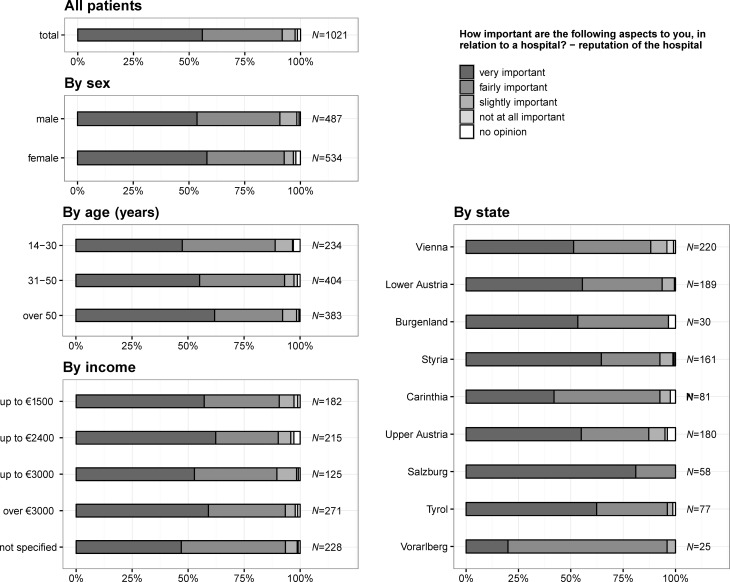
Question 1.1: how important are the following aspects to you, in relation to a hospital? – Reputation of a hospital

In total, 571 (55.9%) citizens assessed the reputation of a hospital as very important (Fig. 1), 312 (60.9%) citizens living in states with a university hospital assessed the reputation of a hospital as very important (states without a university hospital: 259, 52.3%), 163 (31.8%) as fairly important (states without a university hospital: 203, 41.0%), 29 (5.7%) as slightly important (states without a university hospital: 30, 6.1%), 8 (1.6%) as not at all important (states without a university hospital: 3, 0.6%) and 14 (1.4%) had no opinion. The importance of the reputation of a hospital increased with age (*p* = 0.003) and was higher for females (*p* = 0.046). Concerning income per month (*p* = 0.934) no trend could be seen. For states having a university hospital reputation was significantly more important (*p* = 0.016).

### Importance of detailed explanation of treatment as well as information on associated risk factors and possible further treatment

**Fig. 2:**
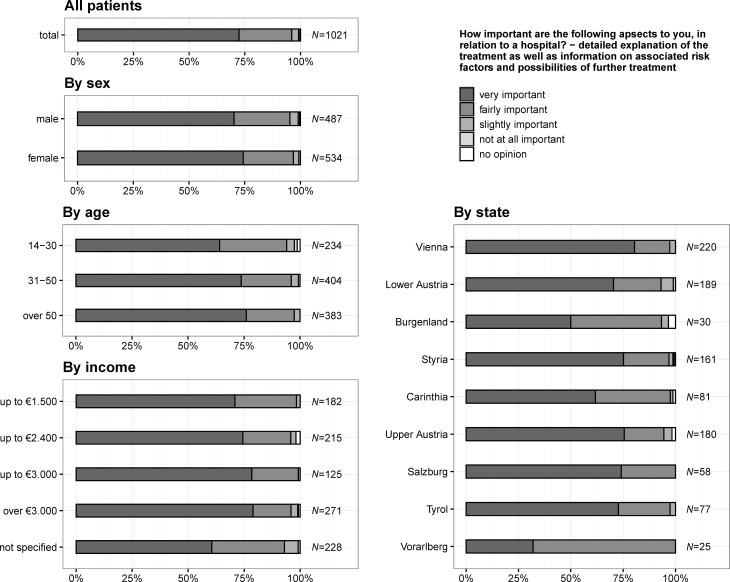
Question 1.2: how important are the following aspects to you, in relation to a hospital? – Detailed explanation of the treatment as well as information on associated risk factors and possibilities of further treatment

In total, 739 (72.4%) citizens stated that detailed explanation of the treatment as well as information on associated risk factors and possible further treatments is very important (see also Fig. 2), 397 (77.1%) citizens living in states with a university hospital answered that is very important (states without a university hospital: 342, 68.4%), 106 (20.6%) that it is fairly important (states without a university hospital: 136, 27.2%), 11 (2.1%) that it is slightly important (states without a university hospital: 20, 4.0%), 1 (0.2%) that it is not at all important (states without a university hospital: 2, 0.4%) and 6 (0.6%) had no opinion. There was no significant difference for sex (*p* = 0.090). The need of detailed explanation increased with age (*p* = 0.006), with the amount of income per month (*p* = 0.047) and was also significantly higher for states having a university hospital (*p* = 0.001).

### Importance of information about patient safety measures

**Fig. 3:**
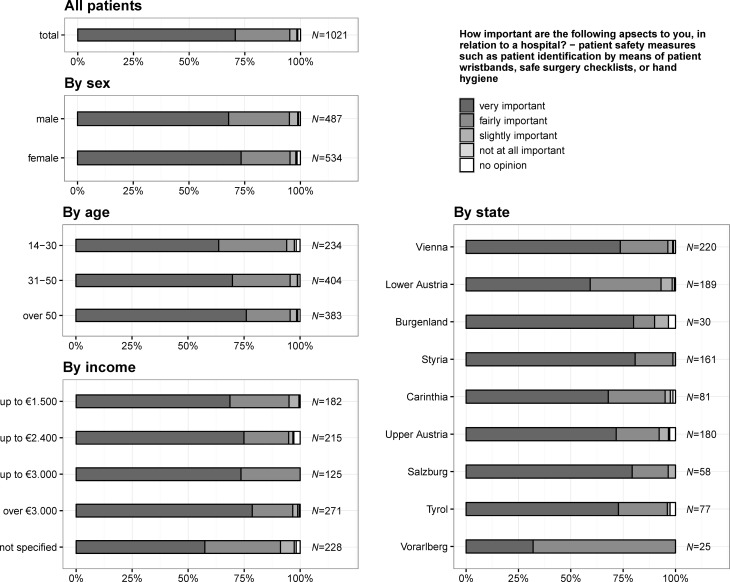
Question 1.3: how important are the following aspects to you, in relation to a hospital? – Patient safety measures, such as patient identification by means of patient wristbands, safe surgery checklists, or hand hygiene

In total, 722 (70.7%) citizens stated that information about patient safety measures, such as patient identification by means of patient wristband, safe surgery, or hand hygiene is very important, 394 (77.0%) citizens living in states with a university hospital answered that it is very important (states without a university hospital: 328, 66.0%), 107 (20.9%) that it is fairly important (states without a university hospital: 143, 28.8%), 10 (2.0%) that it is slightly important (states without a university hospital: 22, 4.4%), 1 (0.2%) that it is not important (states without a university hospital: 4, 0.8%) and 12 (1.2%) had no opinion. The importance of receiving information increased significantly with age (*p* = 0.002), was more important for females (*p* = 0.030), increased by the amount of income per month (*p* = 0.040) and was higher for states having a university hospital (*p* < 0.001) (see also Fig. 3).

### Level of trust/confidence in patient safety within Austrian health care system

**Fig. 4:**
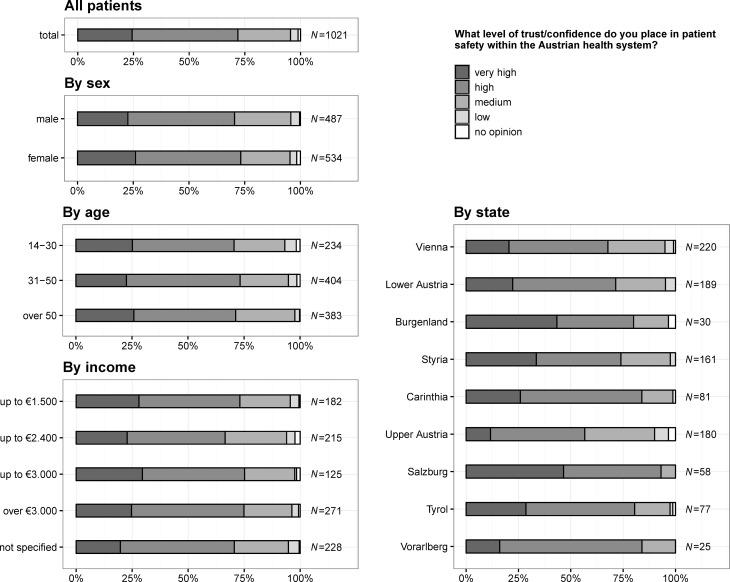
Question 2: what level of trust/confidence do you place in patient safety within Austrian health system?

In total, 249 (24.4%) citizens stated having very high confidence in patient safety (see also Fig. 4), 148 (28.8%) citizens living in states with a university hospital answered that they have a very high trust/confidence in patient safety (states without a university hospital: 101, 20.3%), whereas 236 (46.0%) stated having high (states without a university hospital: 249, 50.1%), 115 (22.4%) medium (states without a university hospital: 126, 25.4%), 14 (2.7%) low (states without a university hospital: 21, 4.2%) confidence in patient safety and 11 (1.1%) had no opinion. There were no significant differences for sex (*p* = 0.092), age (*p* = 0.842) or income per month (*p* = 0.656). For states with university hospitals confidence was significantly higher (*p* = 0.004).

### Importance of receiving information about patient safety measures in your hospital

**Fig. 5:**
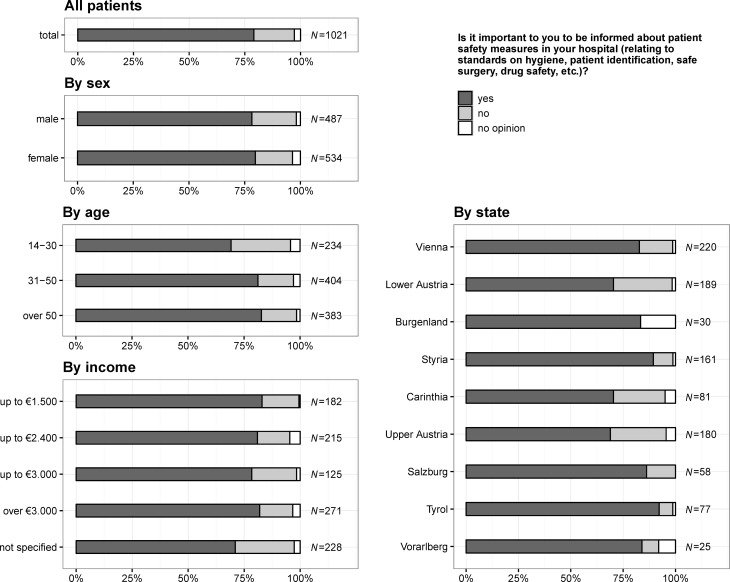
Question 3: is it important to you to be informed about patient safety measures in your hospital (relating to standards on hygiene, patient identification, safe surgery, drug safety, etc.)?

In total, 807 (79.0%) citizens stated that they would like to receive information on patient safety measures in their hospitals (see also Fig. 5), 447 (87.6%) citizens living in states with a university hospital answered that they want to receive information on patient safety measures (states without a university hospital: 360, 74.5%), whereas 63 (12.4%) indicated that they do not need any information (states without a university hospital: 123, 25.5%) and 28 (2.7%) had no opinion. There were no significant differences for sex (*p* = 0.254) and income per month (*p* = 0.924) but increased with age (*p* = 0.001) and was higher for states with university hospitals (*p* < 0.001).

### Complaining if something did not function satisfactorily

**Fig. 6:**
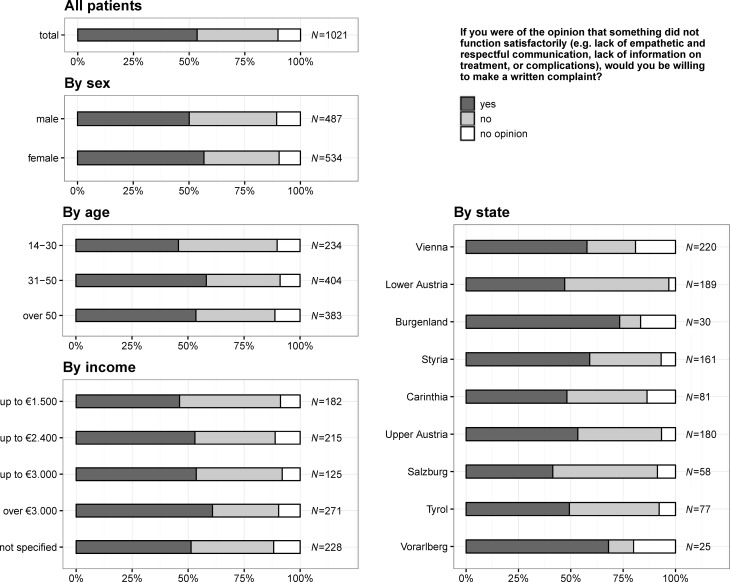
Question 4: if you were of the opinion that something did not function satisfactorily (e. g. lack of empathic and respectful communication, lack of information on treatment, or complications) would you be willing to make a written complaint?

In total, 547 (53.6%) citizens stated that in case something did not function satisfactorily they would address a written complaint to the hospital (see also Fig. 6), 284 (62.8%) citizens living in states with a university hospital answered that they would address a written complaint to the hospital (states without a university hospital: 263, 56.4%), whereas 168 (37.2%) would not complain (states without a university hospital: 203, 43.6%) and 103 (10.1%) had no opinion. There were no significant differences for age (*p* = 0.069) or states having a university hospital (*p* = 0.051), but females (*p* = 0.043) would complain more often and increased with the amount of income per month (*p* = 0.001).

## Discussion

Results of the current survey revealed that 27.4% of interviewed citizens have medium or low trust/confidence in patient safety. Only 24.7% described their trust/confidence in patient safety as very high. Furthermore, more than two-thirds of Austrian citizens indicated that detailed explanation of treatment, information on associated risks and possibilities of further treatment as well as patient safety measures are very important. They also want to be informed about certain patient safety measures in the hospitals. For citizens living in states with a university hospital, the overall importance of “patient safety” seems to be more pronounced. Austrian citizens believe that information on patient safety measures are very important, they want to be involved and the key is communication as they call for better and more detailed information. For citizens living in non-university hospital areas these aspects seem to be less important. Taking these results into consideration for future endeavours, we have to focus more on patient needs so that citizens and patients can feel confident about patient safety in Austrian hospitals.

In 2010 the Special Eurobarometer assessment showed that only 19% of 1001 surveyed Austrians perceived that there is a risk of being harmed during hospital treatment and 79% perceived no risks at all. In the current survey, 27.4% perceived low or medium trust/confidence. Reasons for the differences between both survey results could be diverse. One reason might be that in 2010 citizens were asked about risk whilst in the current survey citizens where asked about their trust/confidence. We also observed that the importance of trust/confidence was significantly higher for states having a university hospital.

As shown by Waterman et al. patients are highly motivated to be an active part in healthcare processes [12]. Our study revealed that Austrian citizens, and especially from states with a university hospital, perceive information on patient safety measures as well as explanation of treatment and information on associated risks as very important. In the past, healthcare professionals perceived that involving patients in the process would be associated with a higher demand on doctors’ time [17], which was probably true in former times. In the meantime, many additional ways to inform patients and thereby get them involved in healthcare process have become available. Social media, smartphone applications, videos for in-house channels in a hospital or leaflets could be supportive. The need of information was also higher within citizens between 14 and 30 years of age and they are the ones who extensively use new media.

According to a Finnish survey, patients between 66 and 75 years of age were most critical concerning treatment safety and 20% reported errors during their hospital stay [18]; however, it became evident that not every patient would report an adverse event. According to the recent survey, 53.6% would address a written complaint to the hospital in case something did not function satisfactorily and this result is in line with results from the Special Eurobarometer [13]. Although the role of patients is changing from being passive recipients to a more active and involved participants in healthcare, it will take some time for patients to believe that they are also an important resource in order to improve unsatisfying processes in healthcare [10].

Concerning reputation, Austrian citizens perceive that the reputation of a hospital is very important and numbers were highest in those states where a university hospital is in place (Tyrol, Salzburg, Styria and Vienna). A study showed that there might be an association between perception of patient safety and reputation of a hospital. Furthermore, a higher reputation was associated with organizations which offered a safer environment and increased with age and income [19]. Our study revealed that the importance of reputation increases with age, is more important for females and is more important for states having a university hospital; however, in contrast to the aforementioned study, no differences emerged with respect to income. Reasons for that might be the fact that healthcare systems are organized in various ways.

## Conclusion

In the past, patient safety initiatives focussed on installing safe procedures and environments in order to predict medical errors without using the important patients’ resources [9]. Reasons for not involving patients in healthcare processes are diverse and healthcare professionals also anticipated certain risks [17]. Our study revealed that patient safety is an important topic for Austrian citizens [1, 9, 20] and they want to be informed and involved. The study also indicates the need to promote patient safety aspects in order to decrease the number of people who are not confident concerning patient safety in Austrian hospitals [21]. Patient involvement is the key to gain further progress in patient safety and has to become an integral part of healthcare strategies. More best practice examples and more data are needed to convince healthcare professionals to do so.
